# Cell-type-resolved colocalisation prioritises *KCNIP2* as a candidate epilepsy drug-target detectable at inhibitory-neuron but not bulk-tissue resolution: a Mendelian randomisation and colocalisation study

**DOI:** 10.3389/fgene.2026.1920945

**Published:** 2026-10-14

**Authors:** Aytac Gul

**Affiliations:** Department of Medical Biology, Tayfur Ata Sökmen Faculty of Medicine, Hatay Mustafa Kemal University, Hatay, Türkiye

**Keywords:** brain expression, cell-type eQTL, colocalisation, drug-target Mendelian randomisation, epilepsy

## Abstract

Most antiseizure medicines act on a limited set of neuronal targets. Approximately a third of patients have treatment-resistant epilepsy, creating a need for mechanistically distinct drug targets. Human genetics can prioritise them, but epilepsy target-discovery has measured expression in plasma or bulk brain tissue and has yielded inconsistent candidate lists. Common-variant association for generalised epilepsy is enriched in neuronally expressed genes, so the cell type measured may determine which druggable targets are detectable. Genes at the genome-wide significant loci of the largest epilepsy meta-analysis were assessed by drug-target Mendelian randomisation (MR). A gene was retained only where the expression and disease signals colocalised on a single causal variant at a posterior probability above 0.8. Each gene was assessed across inhibitory neurons, excitatory neurons, four glial cell types, and bulk cortex, with fine-mapping and directional testing in FinnGen. Of the 23 prioritised genes screened, MR nominated ten and colocalisation retained 2. *KCNIP2* colocalised with generalised epilepsy above the threshold in inhibitory neurons alone. Higher expression was associated with lower risk (posterior 0.95, odds ratio (OR) 0.64, and *p* = 2.5 × 10^−11^), with a weaker, directionally concordant sub-threshold signal in oligodendrocytes (posterior 0.53). The colocalising variant is shared with the neighbouring *CUEDC2* (*r* ^2^ = 0.975), so the two genes cannot be separated on genetic evidence alone. In the bulk cortex, *KCNIP2* carried a strong expression signal mapping to a different causal variant, with no colocalisation. *AP3D1* colocalised across inhibitory neurons, excitatory neurons, and bulk cortex for generalised epilepsy and was associated with increased risk (OR 1.46). Both directions reproduced at the matched generalised-epilepsy endpoint in FinnGen, where *KCNIP2* was protective and *AP3D1* was risk-increasing. The colocalisation did not reproduce there, so FinnGen supports the direction of effect and not the shared-variant evidence. In epilepsy, the cellular compartment in which expression is measured can determine whether a drug-target signal is detected, so analyses restricted to plasma or bulk tissue may miss cell-type-restricted targets such as *KCNIP2*. The demonstration rests on this single locus. Cell-type-resolved colocalisation prioritises *KCNIP2*, which cannot be separated from *CUEDC2*, as a candidate for functional investigation, with *AP3D1* being a genetically supported but pharmacologically intractable second locus.

## 1 Introduction

Epilepsy is among the most common serious neurological disorders. A substantial proportion of epilepsy patients continue to have seizures despite pharmacotherapy (Löscher et al., 2020). The available antiseizure medicines act predominantly on a narrow range of neuronal targets, which constrains the options for these patients and leaves the identification of mechanistically distinct targets an unmet need. Human genetics offers one of the more effective routes to their discovery. Targets with human genetic support show an approximately two-fold higher probability of progression to approval (Nelson et al., 2015; King et al., 2019). The genome-wide signals that carry such support can in turn be mapped onto the genes encoding druggable proteins (Finan et al., 2017). The most recent genome-wide meta-analysis of epilepsy has resolved several common risk loci (Stevelink et al., 2023). However, a risk locus marks a region of the genome rather than the causal gene through which it acts, leaving the target to be identified before the signal can guide therapy. Turning a locus into a target requires determining which gene at the locus carries the effect and the direction in which altering its activity changes risk. Mendelian randomisation (MR) can determine which gene is causal and in which direction. It uses a genetic variant that influences a gene’s expression as an instrument. Since alleles are allocated at random at conception, the resulting estimate of the gene’s causal effect is largely unconfounded by the reverse causation and confounding that limit observational studies. Where the gene encodes a drug target, the MR estimate further predicts the effect of modulating the target with a drug (Schmidt et al., 2020). The validity of that inference, however, depends on the tissue in which expression is measured, given that regulatory variants frequently act in a cell-type-specific manner (Bryois et al., 2022).

Epilepsy is a disorder of neuronal networks in which seizures arise from an imbalance between excitation and inhibition. Most antiseizure drugs target the neuronal ion channels and inhibitory signalling that maintain this balance. Consistent with this, the discovery meta-analysis found generalised-epilepsy-associated genes to be enriched for expression in excitatory and inhibitory neurons rather than in astrocytes, oligodendrocytes, or microglia (Stevelink et al., 2023). That neuronal signal is a gene-set expression enrichment; the same study’s stratified heritability analysis did not separate excitatory from inhibitory neurons, so the cell type within the neuronal compartment is exactly what a bulk analysis leaves unresolved. Therefore, the regulation relevant to generalised epilepsy is largely neuronal. Although glial mechanisms contribute to seizures through other routes (Patel et al., 2019), expression measured in a non-neuronal or mixed compartment may fail to capture.

Studies of epilepsy drug targets have so far measured expression in compartments that may not reflect neuronal regulation. Target-discovery MR for epilepsy has relied on the plasma proteome (Fu et al., 2025; Gui et al., 2025) or on bulk brain tissue, with the most recently in a multi-scale integration that combined epilepsy GWAS with bulk and single-cell eQTL in which the principal target, *FGFR3*, was localised to an astrocyte-centred signalling loop (Zhong et al., 2026).

An astrocyte-centred principal target is difficult to reconcile with the neuronal regulation of generalised epilepsy. Different studies nominate different genes, and the same instability recurs in drug-target screens of other brain disorders run at a single resolution (Li et al., 2023; Liao et al., 2025). This disagreement has a structural explanation. Protein levels measured in plasma do not reflect how the same gene is regulated in neurons. Bulk tissue averages expression across every cell type, so a variant acting in a single neuronal population can be diluted below detection while a variant prominent in bulk may be driven by the non-neuronal majority. A second source of error is independent of compartment. Two nearby variants inherited together can produce a cis-MR association even when one controls the gene and a different one affects disease, a violation of the MR assumptions that only colocalisation can resolve from a single shared causal variant (Giambartolomei et al., 2014; Zuber et al., 2022). No study has directly tested whether the inconsistency between candidate lists arises from measuring expression in the wrong compartment, so that a gene detectable in one cell type is missed in another.

To address this, the genes prioritised at the genome-wide significant loci of the largest epilepsy meta-analysis were evaluated in two steps (Figure 1). First, MR proposed the genes whose expression was associated with epilepsy risk. Colocalisation then tested whether the expression and disease signals at a locus were driven by a single shared causal variant. A gene was retained only when both steps agreed, which excludes candidates supported by MR but not by a shared causal variant. Each gene was assessed across inhibitory neurons, excitatory neurons, four glial cell types, and bulk cortex. FinnGen provided a directional test, and a candidate reported earlier from plasma-proteome MR was re-examined in plasma. The screen returned two genes that illustrate the two outcomes. *KCNIP2* colocalised with epilepsy only in inhibitory neurons and not in bulk cortex, where its expression and the disease associations mapped to separate variants, whereas *AP3D1* colocalised in neurons and in bulk tissue alike.

**FIGURE 1:**
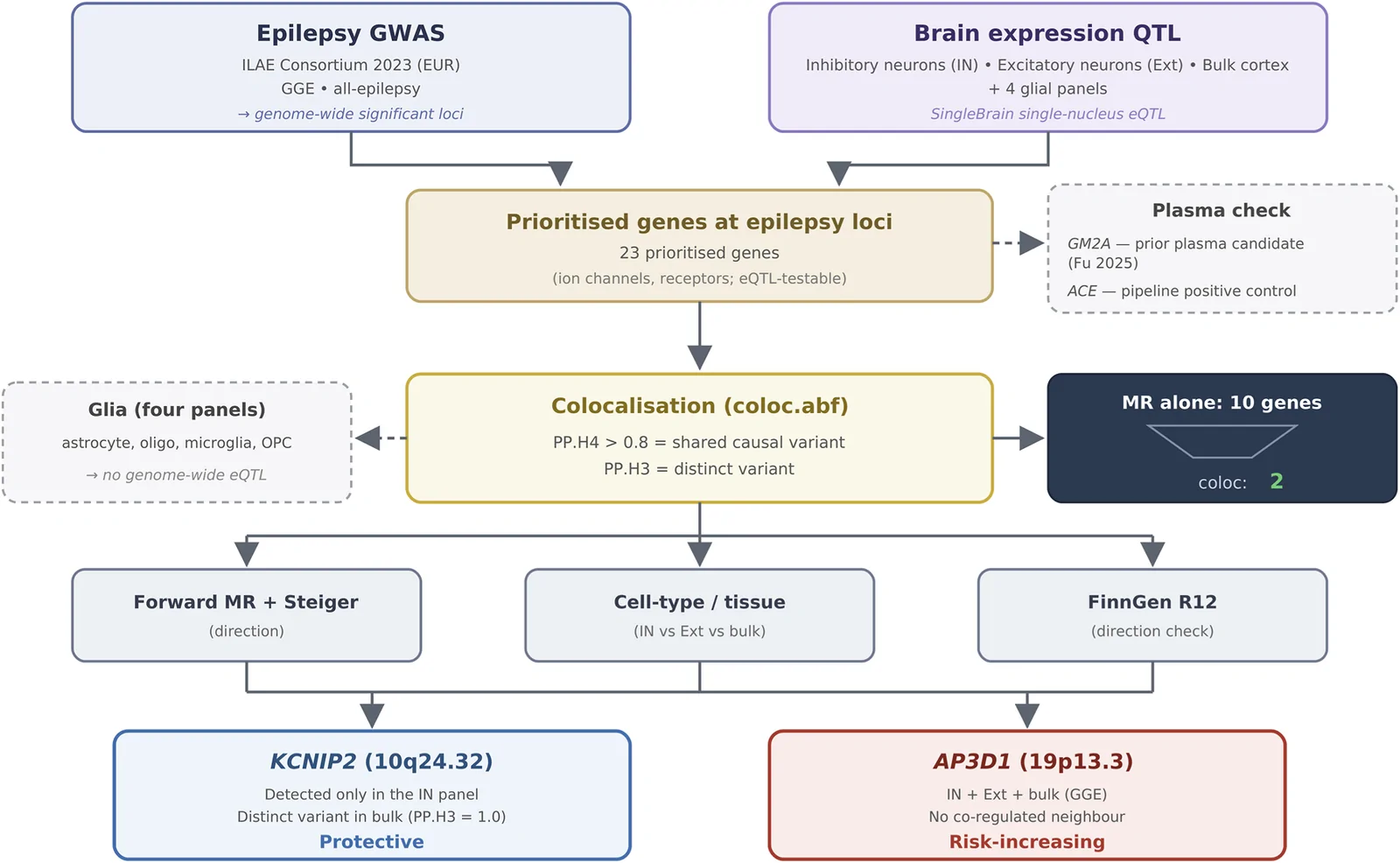
Study design of the cell-type-resolved drug-target screen. Genes prioritised at the genome-wide significant loci of the ILAE epilepsy meta-analysis enter a two-step screen. Forward MR first proposes the genes whose genetically predicted expression is associated with epilepsy. Colocalisation then tests whether the expression and disease signals at each locus are driven by a single shared causal variant, retaining a gene only when both steps agree. Each gene is assessed in inhibitory neurons, excitatory neurons, and bulk cortex, with four glial panels, directional testing in FinnGen, and a plasma check of a previously reported candidate in deCODE.

## 2 Materials and methods

### 2.1 Study design and candidate genes

Drug-target MR was anchored in the inhibitory-neuron panel, and colocalisation was then applied separately within each brain-cell type. The exposure was the expression of the genes prioritised at established epilepsy risk loci, instrumented by their cis-acting expression quantitative trait loci. Inhibitory neurons were the primary exposure panel, and excitatory neurons were analysed in parallel. Generalised-epilepsy association is enriched in neuronally expressed genes, but this enrichment does not distinguish inhibitory from excitatory neurons.

These genes were also tested in four glial cell types and in bulk cortex, so each gene was compared across resolutions. The outcome was epilepsy, defined as genetic generalised epilepsy (GGE), and as all epilepsy in the largest genome-wide meta-analysis, with directional testing in FinnGen. Candidates were confined to the genes prioritised at the genome-wide significant loci of that meta-analysis, since colocalisation can identify a shared causal variant only where the disease association already carries a regional signal. The design is, therefore, a post-GWAS locus-to-gene prioritisation within established epilepsy loci, not an independent discovery or replication of the disease associations themselves. The loci were selected based on their association with the same outcome, so outcome associations are enriched by construction. MR significance is, therefore, read as a prioritisation step, not as an independent layer of disease evidence**.** The ILAE Consortium on Complex Epilepsies named a single most likely implicated gene at each such locus using a 10-criterion scoring scheme. At one locus (1q43), the scheme prioritised two genes, *RYR2* and *CHRM3*, of which *RYR2* was carried forward, giving 23 genes from the 24 prioritised genes. A gene was included if it was protein-coding and brain-expressed, with a measurable cis-eQTL in at least one panel. Supplementary Table S1 lists every prioritised gene and the criterion that included or excluded it, giving the full denominator for the cell-type-specificity of the results.

*CHRM3* was analysed in excitatory neurons, where it carries a genome-wide significant cis-eQTL. The screen is anchored in inhibitory neurons by design, and a gene reaching instrument strength only in excitatory neurons is tested there rather than dropped. The MR estimate uses the highest-*F* excitatory variant present in ILAE3, since none of the four variants passing *p* < 5 × 10^−8^ in the excitatory panel is present there.

### 2.2 Data sources

Summary statistics for GGE and for all types of epilepsy were taken from the largest genome-wide meta-analysis of the ILAE Consortium on Complex Epilepsies (Stevelink et al., 2023). The European-ancestry subset was used to match the ancestry of the expression data. The control samples are partially shared across the epilepsy phenotypes in that meta-analysis. Both were also analysed in FinnGen release 12 (Kurki et al., 2023), taking its generalised epilepsy endpoint as the closest match to GGE and its all-epilepsy endpoint for the larger case count. Cell-type cis-eQTL were taken from SingleBrain, a single-nucleus RNA-sequencing brain eQTL meta-analysis of four European-ancestry cohorts combined with a linear mixed-model random-effects method (Jang et al., 2026).

SingleBrain integrates the cohorts of Bryois et al., 2022; Fujita et al., 2024; Mathys et al., 2023; and Gabitto et al., 2024. It provides cis-eQTL for inhibitory neurons, excitatory neurons, and four glial cell types. Bulk cortex cis-eQTL were taken from MetaBrain (de Klein et al., 2023), an independent multi-cohort eQTL meta-analysis of European cortical tissue. This resource shares no samples with the single-nucleus data, so a colocalisation obtained in bulk cortex is independent support for the expression signal.

Effect sizes and standard errors from the two eQTL resources were used. Bulk-cortex effects were converted to a per-standard-deviation scale, so they were comparable with the other datasets. Each per-allele effect and its standard error were derived from the z statistic, the effect-allele frequency *f*, and the sample size N using the standard approximation *β* = z/√(2*f*(1 - *f*)(*N* + z^2^)), with the standard error being the reciprocal of that square-root term. The expression-scale variance required by the colocalisation likelihood was estimated from the allele frequency and sample size where eQTL effects were not already standardised. Cis protein QTL for *GM2A* were taken from the deCODE plasma proteomics resource (Ferkingstad et al., 2021), which measured the plasma proteome by SomaScan in an Icelandic cohort. The same resource provided the cis-pQTL for *ACE*, included as a positive control that a strong plasma cis-pQTL instrument can recover correctly. All datasets and their sample sizes are summarised in Table 1. The full list of datasets with versions and accession identifiers is given in Supplementary Table S2.

**TABLE 1:** Genetic datasets used in the analysis.

Dataset	Role	Phenotype	Ancestry	Genome build	Cases or donors	Controls	Effective N (per-variant median)	Source
ILAE Consortium (Stevelink et al., 2023)	Outcome (discovery)	Genetic generalised epilepsy (GGE)	European	GRCh37	6,952	42,434	23,281	https://www.epigad.org/
ILAE Consortium (Stevelink et al., 2023)	Outcome (discovery)	All epilepsy	European	GRCh37	27,000	42,434	64,843	https://www.epigad.org/
FinnGen release 12 (Kurki et al., 2023)	Outcome (replication)	Generalised epilepsy (phenotype-matched)	Finnish European	GRCh38	1,690	Population controls (FinnGen R12 ≈ 500,000)	Not reported	https://www.finngen.fi/
FinnGen release 12 (Kurki et al., 2023)	Outcome (replication)	All epilepsy (G6_EPLEPSY)	Finnish European	GRCh38	15,645	Population controls (FinnGen R12 ≈ 500,000)	Not reported	https://www.finngen.fi/
SingleBrain single-nucleus brain eQTL (Jang et al., 2026; Bryois et al., 2022; Mathys et al., 2023; Gabitto et al., 2024)	Exposure (primary)	Cell-type cis-eQTL: inhibitory neurons, excitatory neurons, and four glial panels	European	GRCh38	983 donors	Not applicable	Not applicable	Zenodo 10.5281/zenodo.14908182
MetaBrain bulk cortex eQTL (de Klein et al., 2023)	Exposure (resolution contrast)	Bulk cortical cis-eQTL	European	GRCh38	2,000 donors	Not applicable	Not applicable	https://www.metabrain.nl/
deCODE plasma proteomics (Ferkingstad et al., 2021)	Exposure (plasma check)	Plasma cis-pQTL for *GM2A*, with *ACE* as a pipeline positive control	European (Icelandic)	GRCh38	35,559	Not applicable	Not applicable	https://www.decode.com/summarydata/
1000 Genomes Phase 3, European subset (Auton et al., 2015)	Linkage-disequilibrium reference	Not applicable	European	GRCh37	503	Not applicable	Not applicable	https://www.internationalgenome.org/

A protein-level test of the *KCNIP2* finding is not currently possible. *KChIP2* is an intracellular potassium-channel interacting protein. It is absent from available brain and cerebrospinal fluid pQTL resources. The plasma proteomic resource used here as a positive control carries no cis-pQTL for it. The proposed causal chain rests on transcript abundance. Whether the effect propagates to the protein level in inhibitory neurons is untested. This is stated as a limitation. The abstract and conclusion frame the corresponding claims at the level of expression, not protein activity.

### 2.3 Mendelian randomisation

Cis instruments were variants within 500 kb of the transcription start site. The F statistic was computed as the square of the variant z statistic, so the instrument strength remained numerically stable for variants whose reported *p-*value underflowed to zero. Allele harmonisation used rsID, since the datasets differed in genome build. Harmonisation is reported in full as the datasets differ in genome build. Variants were matched on rsID rather than position. SingleBrain and FinnGen used GRCh38. ILAE3 and the 1000 Genomes reference use GRCh37. Effect alleles were aligned by comparing the eQTL effect allele with the outcome’s first allele. The outcome effect sign was flipped where they differed. This flip applied to the bulk-cortex comparison at rs10883695. Its supplied effect allele is G against A in the single-cell panels. Palindromic variants, those with A/T or C/G alleles where the strand cannot be resolved from the allele codes alone, were not excluded. Both datasets report alleles on the forward strand of the same assembly. They are matched on rsID. This class is handled by the same effect-allele comparison as every other variant. A frequency-based strand check was not applied. Palindromic variants make up 13.9% of shared variants at 10q24.32. Removing them changes the *KCNIP2* colocalisation posterior from 0.950 to 0.959 across 1,782 variants instead of 2,054. This choice does not affect the result (Supplementary Table S3). One instrument, *RORB* (rs4351477, G/C), is palindromic. It is an exploratory instrument already excluded from the strict analysis. Multi-allelic sites are absent by construction.

Each panel reports one bi-allelic record per rsID, and the merge produced no duplicated identifiers. Allele frequencies were checked only as a validity filter. Variants with a frequency of zero or one were dropped. Frequency was not used to infer strand. Variants absent from the outcome were replaced by the highest-*F* cis-variant present, as described above. No positional liftover was performed at any step. No coordinate-conversion error can enter the harmonised estimates. Two supplementary tables report an *F* statistic for the same gene, and they refer to different variants. Supplementary Table S4 reports the F of the variant used as the MR instrument. Supplementary Table S5 reports the F of the strongest cis variant for that gene in the panel. Where the instrument is not the panel lead, the two differ, as for *SCN1A* at 125.3 against 223.3, and both values are correct for what they describe. The full provenance of every *F-*value is given in Supplementary Table S6.

Whether the 16 non-colocalising genes also fail to colocalise in excitatory neurons was determined from the raw panel data rather than from the stored summary. Four features of the data bound what the analysis can claim. Independent signals per region were counted as genome-wide significant variants pruned at *r* ^2^ < 0.1 in the 1000 Genomes European reference. LD-reference sensitivity was assessed by splitting the 503-sample European reference in half and recomputing the correlations. Credible sets were computed at 0.95 coverage. Purity and convergence diagnostics were not retained by the analysis, so credible-set width is the reported diagnostic. The SingleBrain eQTL panels rest on 983 donors, and the same donor set contributes to every cell type. The panels, therefore, differ in the number of nuclei assigned to each cell class rather than in donor identity.

Two controls bound the interpretation. *ACE* is a control for plasma pQTL extraction and instrument recovery, not a positive control for colocalisation. It confirms that a strong cis-pQTL is retrieved and aligned correctly, which is what makes the *GM2A* null interpretable. The colocalisation implementation was checked against the reference R implementation of coloc.abf on both retained loci. The variable reported as nSNP throughout the supplement is the number of variants shared between the expression and outcome datasets inside the colocalisation window. It is not the number of MR instruments, which is one per gene, except where stated.

Two admission rules were applied, which are reported separately. A gene entered the primary analysis when its instrument reached *p* < 5 × 10^−8^. This corresponds to *F* = 29.7 on the identity *F* = z2. A gene entered an exploratory tier when its strongest cis variant fell short of that threshold. It still needed to reach *F* ≥ 10. The exploratory tier is reported as exploratory throughout. It is not counted towards the primary result. *CACNA2D2* (*F* = 6.2, *p* = 0.013) and *BCL11A* (*F* = 9.1, *p* = 2.6 × 10^−3^) failed both rules. They are reported as weak instruments whose estimates are uninterpretable. Of the 18 genes with a testable cis instrument, eight met the primary threshold, eight fell in the exploratory tier, and two were weak. The instrument was the cis variant with the highest F statistic among those present in the outcome dataset. For 11 genes, this variant was also the strongest cis variant in the panel. For seven genes, the panel lead was absent from ILAE3.

The highest *F-*variant present was used instead. This substitutes for an untyped lead. It is not selected by physical distance. The instrument table reports the exposure beta and standard error for every instrument. It also shows the exposure *p-*value and the *F-*statistic. Further columns show the minor allele frequency in the 1000 Genomes European reference. Other columns show whether the variant is the panel lead or a substitute, along with the identity of the panel lead. The table also states whether that lead was present in ILAE3. It provides the squared correlation between the instrument and the panel lead in the same reference. These correlations are low for most substitutions; *r* ^2^ = 0.002 for *SCN1A*, 0.006 for *CACNA2D2*, 0.003 for *BCL11A*, 0.009 for *SUGCT*, and 0.022 for *SCN8A*. One is high; *r* ^2^ = 0.894 for *KCNN2*. A substituted instrument generally interrogates a different cis signal from the panel lead. It is not a correlated proxy for it. The panel lead for *POU3F3* is absent from the reference panel. Its correlation was not computed.

Palindromic variants were resolved by allele frequency and excluded when the minor allele frequency (MAF) exceeded 0.42. Where a gene resolved to a single independent cis signal after clumping at *r* ^2^ < 0.001, the causal effect was the lead-variant Wald ratio. For genes with more than one cis variant passing the F threshold, a correlated inverse-variance-weighted (IVW) estimate over the wider cis set was computed in addition (Burgess et al., 2016) using the European reference linkage-disequilibrium matrix.

This correlated estimate is sensitive to matrix regularisation when the cis variants are highly correlated, so it served only to corroborate the direction of effect, with the lead-variant Wald ratio retained as the primary estimate throughout (Supplementary Table S7). The correlation matrix for those instruments is given in Supplementary Table S8. Direction and magnitude were estimated by forward MR, as an odds ratio (OR) per standard-deviation increase in genetically predicted expression. The 95% confidence intervals (CIs) were derived from the OR and its *p-*value (z = Φ^−1^(1 - p/2), leading to a standard error of |ln OR|/z). For the strong instruments used here, with F above 29 at both retained genes, the additional variance contributed by the eQTL estimate is negligible, so the exposure-side standard error was not separately propagated. Reverse causation was assessed by Steiger filtering (Hemani et al., 2017) and is reported for every tested gene in Supplementary Table S9.

The Steiger results were treated as supportive but not decisive, since the test favours the exposure-to-outcome direction when the outcome is weakly powered. Heterogeneity was assessed by Cochran Q and the directional-pleiotropy intercept. Multi-instrument estimates were additionally checked by leave-one-out analysis. MR *p-*values were corrected by the Benjamini–Hochberg procedure (Benjamini and Hochberg, 1995) across the full family of gene-by-phenotype tests, restricted to gene–phenotype pairs with a valid cis instrument. The generalised-epilepsy and all-epilepsy analyses share controls, so their tests are positively correlated, a condition under which the Benjamini–Hochberg procedure remains valid and is, if anything, conservative. Colocalisation posteriors were not entered into this frequentist correction, as they are Bayesian posteriors and not null-hypothesis *p-*values.

### 2.4 Colocalisation and fine-mapping

Colocalisation between each gene cis-eQTL and the epilepsy association used the approximate Bayes factor method under a single-causal-variant assumption (Giambartolomei et al., 2014). Each test was performed in a 1-Mb window centred on the gene to capture regional linkage disequilibrium. Approximate Bayes factors were computed from each variant’s effect and standard error. The prior standard deviation was 0.2 for the binary epilepsy trait and 0.15 for the quantitative expression trait, with the latter scaled by the estimated expression standard deviation. Prior probabilities were set to 1 × 10^−4^ for a variant associated with expression alone, 1 × 10^−4^ for a variant associated with epilepsy alone, and 1 × 10^−5^ for a variant associated with both traits. Since these approximate Bayes factors were derived from reported effects and standard errors, the resulting posteriors do not depend on the case fraction of the epilepsy datasets. The posterior probability of a single shared causal variant (PP.H4) above 0.8 was taken as colocalisation. A high posterior probability of two distinct causal variants (PP.H3) was taken as positive evidence against a shared mechanism, which is distinct from a simple lack of statistical power. Three further analyses tested stability and regional specificity. First, the sensitivity of PP.H4 to the shared-variant prior was assessed across five values spanning two orders of magnitude (×110^−6^ to 1 × 10^−4^). Second, each colocalising gene was tested against every other gene with a testable cis-eQTL in its locus to establish whether the candidate was the regional driver.

Third, co-regulation between a candidate and its neighbours was tested by colocalising the two genes’ expression signals and reporting the linkage disequilibrium between their lead expression variants, since a shared regulatory variant cannot be resolved to one gene on genetic evidence alone. The single-variant assumption was tested by repeating colocalisation with a fine-mapping-based method (Wallace, 2021) at the two colocalising loci, *KCNIP2* and *AP3D1*, for GGE. Each trait was fine-mapped with the sum of single effects (SuSiE) model (Wang et al., 2020) using the European reference, allowing up to 10 causal signals per region and a credible-set coverage of 0.95. For every gene that was MR-significant but colocalisation-negative with regional eQTL data, colocalisation was also repeated after masking the primary shared peak and its flanking 250 kb. This removes the dominant signal and tests whether a second independent shared variant remains.

The epilepsy trait was fitted in the case-control mode at the European GGE case fraction of approximately 0.14. Summary-statistic fine-mapping with a limited external reference is sensitive to reference mismatch, so the credible sets are reported as a confirmatory check with any instability noted, and the confirmatory weight is placed on the colocalisation posterior rather than on credible-set width. Shared *versus* distinct causal variants were examined further by approximate conditional analysis, re-evaluating each gene’s residual epilepsy association after conditioning on the lead shared variant. A gene was defined as a credible target only when both criteria were satisfied, a PP.H4 above 0.8 and a Benjamini–Hochberg false-discovery rate (FDR) below 0.05.

### 2.5 Cross-resolution comparison and FinnGen testing

The epilepsy outcome and the colocalisation window were held fixed across the inhibitory-neuron, excitatory-neuron, and bulk-cortex panels, with each panel contributing its own cell-type cis-eQTL, so the colocalisation posterior could be compared directly across resolutions. The inhibitory-neuron instrument was retained for the MR estimate throughout rather than re-derived per panel. Each candidate was also screened for a cis-eQTL in the four glial panels, where absence was defined as no variant reaching *p* < 5 × 10^−8^ in the cis window. Testing of the two credible targets in FinnGen required directional concordance and nominal significance at the phenotype-matched generalised-epilepsy endpoint, which is smaller than the discovery dataset and not expected to carry a genome-wide signal at these loci.

### 2.6 Software and data availability

Colocalisation by approximate Bayes factor and the Wald-ratio MR were implemented in Python with NumPy and pandas. The approximate Bayes factor posteriors from this implementation were reproduced with the standard coloc R package at both retained loci, agreeing to within 0.0002 of the posterior (Supplementary Table S10). SuSiE fine-mapping and SuSiE-based colocalisation were implemented in R with the coloc and susieR packages. The linkage-disequilibrium reference was the 1000 Genomes Phase 3 European subset (Auton et al., 2015). Full package versions are listed in the code repository. All input summary statistics are publicly available, as listed in Table 1.

## 3 Results

### 3.1 Two prioritised genes colocalise with epilepsy in inhibitory neurons

Twenty-three prioritised epilepsy-locus genes were screened for colocalisation between their inhibitory-neuron cis-eQTL and the two epilepsy associations (Figure 2). Two genes cleared a shared-variant posterior (PP.H4) of 0.8, with *KCNIP2* at 0.95 and *AP3D1* at 0.94. The remaining 21 resolved into three patterns. Eight carried both a disease and an expression signal at the locus but on distinct variants, a high posterior for two causal variants (PP.H3 above 0.5), and among them were *SCN1A*, *SCN8A*, *RMI1*, and *GLS*, all at PP.H3 of 0.98 or higher. This configuration produces an MR association without colocalisation and it includes *SCN1A* and *SCN8A*, where epilepsy risk is carried by rare coding variation rather than the common regulatory variation tested. Nine had no regional signal able to support a shared variant, with both posteriors low. Four could not be tested, lacking a measurable cis-eQTL in inhibitory neurons (*STAC*, *SPOCK1*, *ARHGEF15*, and *TMPRSS15*).

**FIGURE 2:**
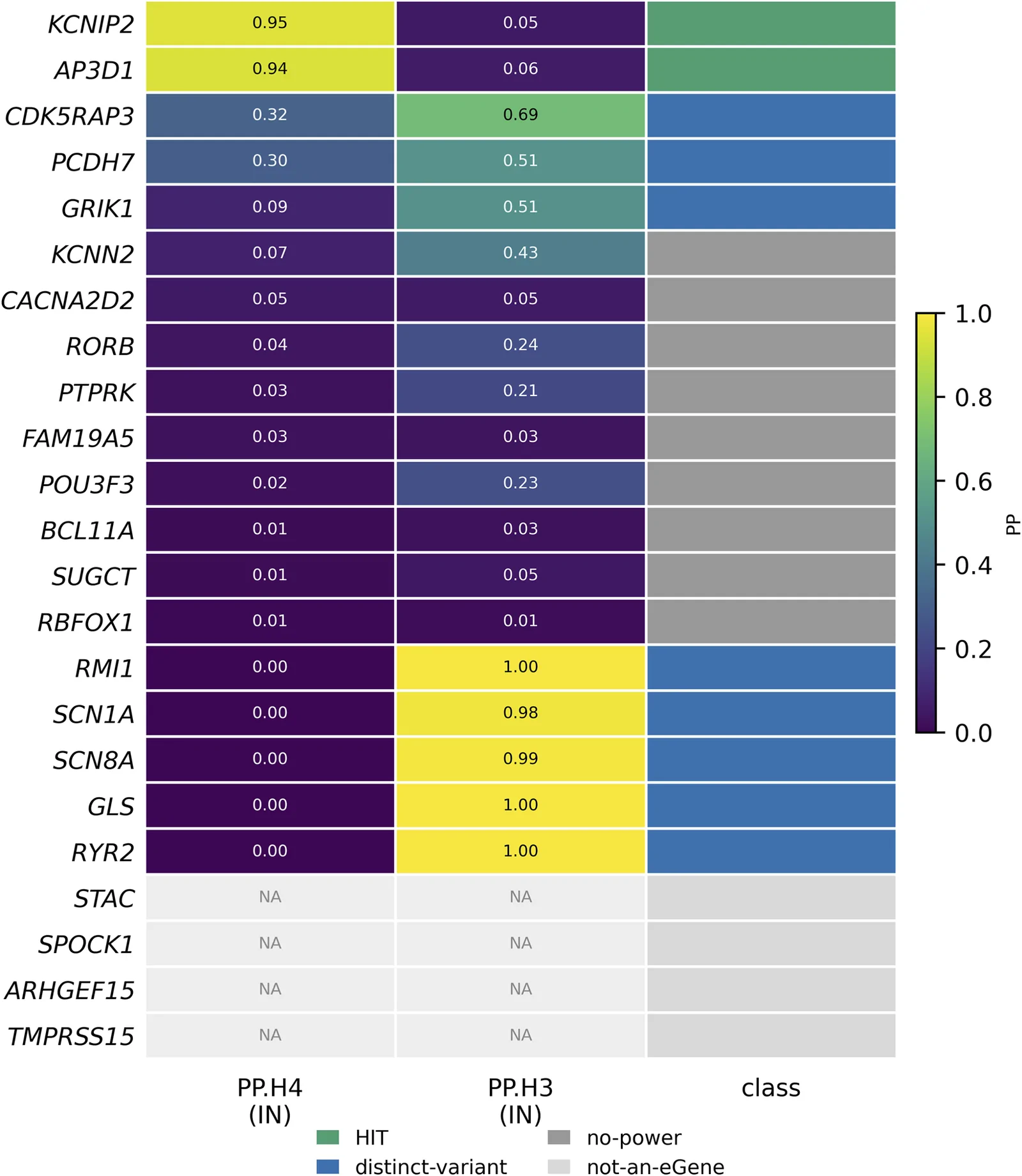
Colocalisation screen across the 23 prioritised genes. Heatmap of the shared-variant posterior (PP.H4) and distinct-variant posterior (PP.H3) from colocalisation of each gene’s inhibitory-neuron cis-eQTL with generalised epilepsy, for the 23 screened genes ordered by PP.H4. A third column marks each gene’s class as a hit (PP.H4 > 0.8), a distinct-variant locus, a no-power locus, or a gene lacking a measurable inhibitory-neuron cis-eQTL.

### 3.2 Cell-type-resolved colocalisation at the two credible loci

At 10q24.32, *KCNIP2* colocalised with both epilepsy phenotypes in inhibitory neurons (posterior 0.95 for GGE and 0.94 for all epilepsy) and had the highest colocalisation posterior across the region (Figure 3A; Supplementary Table S11). The locus is pleiotropic. It carries many strong expression signals, several at higher instrument strength than *KCNIP2*, yet only *KCNIP2* colocalised with epilepsy and the neighbouring *CUEDC2* approached the threshold. *CNNM2* and *NT5C2* mapped to distinct regional signals, at posteriors below 0.04. A colocalisation at this locus must, therefore, be interpreted variant by variant. *KCNIP2* cannot be separated from *CUEDC2* on genetic evidence alone, since the two share a single inhibitory-neuron regulatory variant, with their strongest cis-eQTL variants in near-complete linkage disequilibrium (*r* ^2^ = 0.975, Supplementary Table S12). Conditioning on the shared variant removed the epilepsy association for both. *KCNIP2* carries the larger expression effect and statistical weight, but *CUEDC2* cannot be excluded.

**FIGURE 3:**
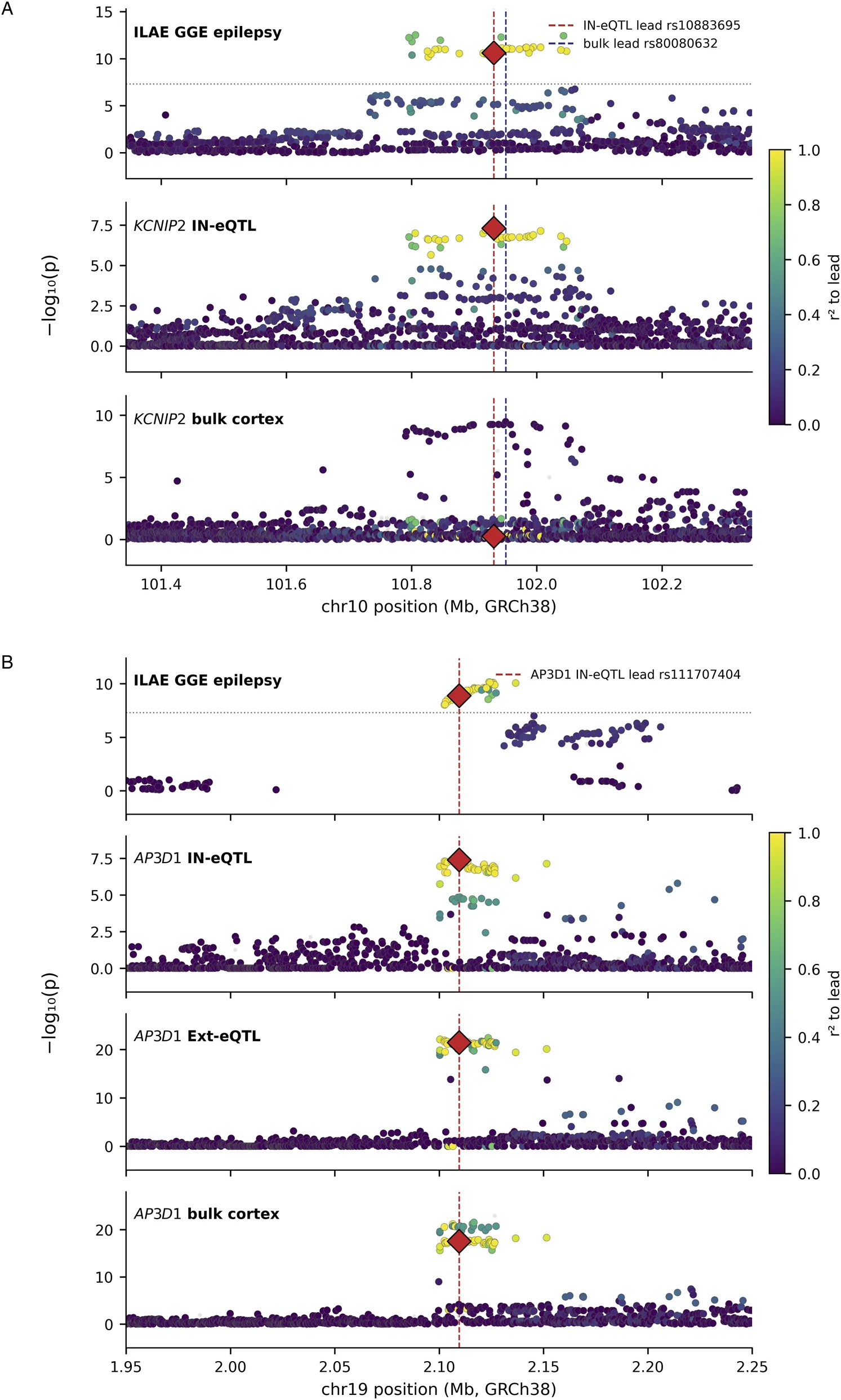
Regional colocalisation at the two credible loci. Stacked regional association plots aligning the epilepsy signal with cell-type and bulk expression. **(A)** 10q24.32, where the inhibitory-neuron *KCNIP2* signal aligns with epilepsy, while the bulk-cortex peak maps to a distinct variant. **(B)** 19p13.3, where the *AP3D1* signal aligns across inhibitory neurons, excitatory neurons, and bulk cortex. Points are coloured by linkage disequilibrium to the lead.

Genetic evidence at 10q24.32 does not separate *KCNIP2* from *CUEDC2*. The two lead variants sit in near-complete linkage disequilibrium (*r* ^2^ = 0.975). *CUEDC2* returns a colocalisation posterior of 0.770 against generalised epilepsy. This is below the 0.8 threshold used throughout but is not evidence against a *CUEDC2* effect. A posterior of 0.770 is sub-threshold, not a rejection. The gap between it and the 0.950 obtained for *KCNIP2* is small relative to the uncertainty in either estimate. Fine-mapping is equally unable to resolve the locus. SuSiE returns a single credible set of 25 variants for *KCNIP2*. Its maximum posterior inclusion probability is 0.111. SuSiE also returns a single credible set of 33 variants for *AP3D1*. Its maximum probability is 0.057. No individual variant approaches the concentration needed for a single-gene claim. *KCNIP2* is named as the candidate at this locus on the strength of its cell-type-restricted regulation and channel biology. This is not because *CUEDC2* has been excluded. Every statement about *KCNIP2* in this work should be read as a statement about the *KCNIP2*/*CUEDC2* locus. At 19p13.3, *AP3D1* colocalised with GGE in inhibitory neurons, excitatory neurons, and bulk cortex (posteriors 0.94, 0.94, and 0.92) and had the highest colocalisation posterior among the 95 genes with a testable cis-eQTL across the locus, with no co-regulated neighbour (Figure 3B). For all epilepsy, it did not colocalise in any panel (posteriors 0.29, 0.25, and 0.08), so its colocalisation across the three panels is specific to the genetic generalised epilepsy.

Thirteen variants in the *AP3D1* cis window reach genome-wide significance, so the single-instrument estimate was checked for independent signal rather than assumed. All are in complete linkage disequilibrium with the lead in the 1000 Genomes European reference (*r* ^2^ = 1.000 for every pair). Effect estimates are numerically indistinguishable, ranging from −0.2081 to −0.2087, with a standard error of 0.038 throughout. They represent one signal recorded 13 times, not 13 instruments (Supplementary Table S13). An inverse-variance weighted estimate across them divides by an effective sample size 13 times too large and reports a spuriously narrow confidence interval. A correlated-weights estimator with this LD matrix reduces to the single-variant estimate, so the lead-variant estimate is reported. The colocalisation posterior of 0.939 uses the whole regional signal and is unaffected either way.

### 3.3 Drug-target MR gives the two genes opposite directions

Forward MR estimated the direction of effect for each gene (Figure 4). Higher inhibitory-neuron *KCNIP2* expression was associated with lower epilepsy risk at an OR of 0.64 (*p* = 2.5 × 10^−11^ for GGE), consistent with both phenotypes (Supplementary Table S14). The correlated IVW estimate over the wider cis set agreed in direction. Higher *AP3D1* expression was associated with higher GGE risk at an OR of 1.46 (95% CI 1.29 to 1.64, *p* = 1.3 × 10^−9^) in inhibitory neurons. For all epilepsy, the *AP3D1* effect was weaker, at an OR of 1.12, matching its loss of colocalisation.

**FIGURE 4:**
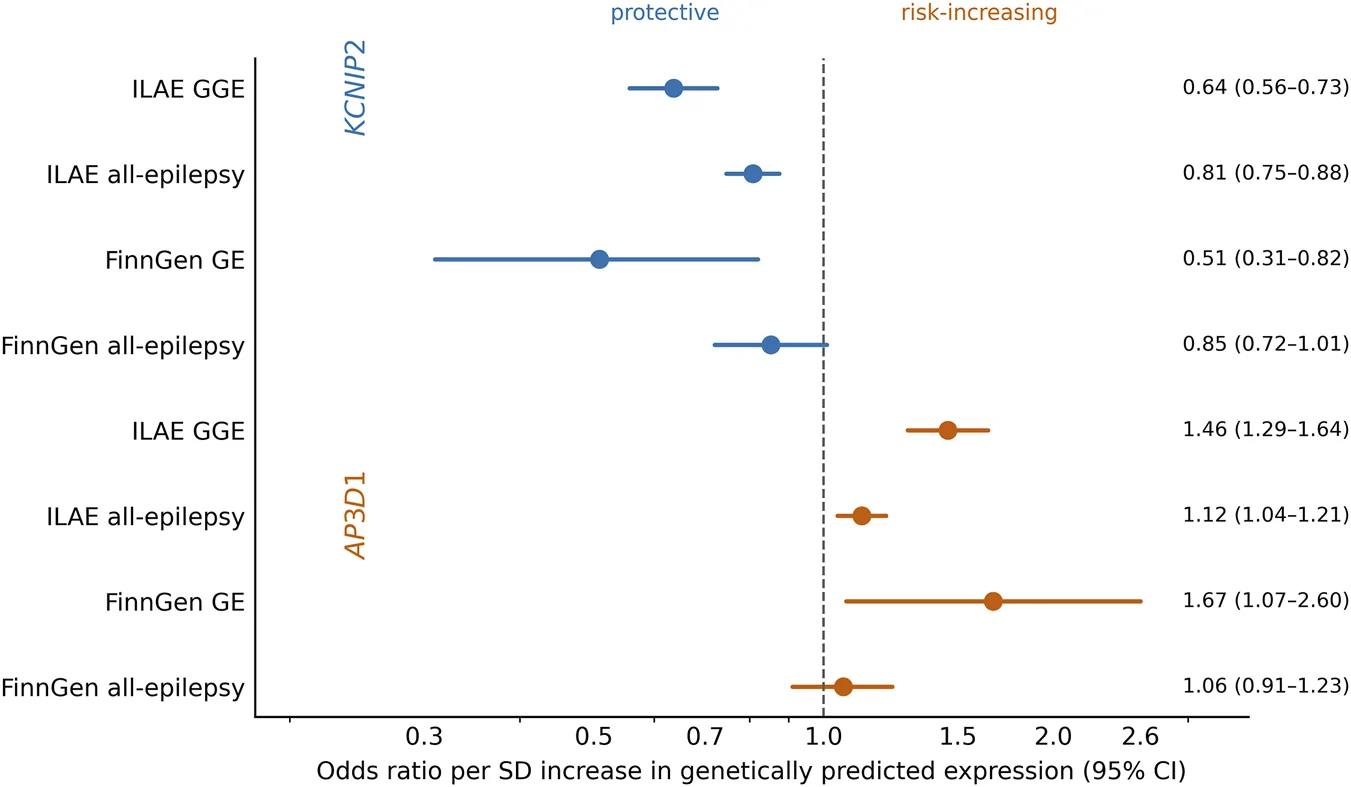
Drug-target Mendelian randomisation estimates with directional testing in FinnGen. Forest plot of the forward MR ORs for *KCNIP2* and *AP3D1*, expressed per standard-deviation increase in genetically predicted expression. Estimates are shown for the two discovery phenotypes (ILAE genetic generalised epilepsy and all epilepsy) and the matched FinnGen endpoints, with 95% confidence intervals. *KCNIP2* is protective and *AP3D1* is risk-increasing in both the discovery and FinnGen datasets.

Both genes were tested in FinnGen, an independent biobank in a separate population (Supplementary Table S15). Both directions reproduced at the phenotype-matched generalised-epilepsy endpoint and reached nominal significance, with *KCNIP2* protective at an OR of 0.51 (*p* = 6.0 × 10^−3^) and *AP3D1* risk-increasing at an OR of 1.67 (*p* = 2.4 × 10^−2^). The discovery lead variant for *KCNIP2* was again the lead in FinnGen. Colocalisation did not reproduce in FinnGen, where the posterior fell on the expression-only hypothesis. FinnGen, therefore, supports the direction of effect but does not independently replicate the colocalisation.

The FinnGen analysis tested two genes at two endpoints, giving four MR tests and a Bonferroni threshold of *p* = 0.0125. *KCNIP2* meets it at the matched generalised-epilepsy endpoint (GE, 1,690 cases, *p* = 6.0 × 10^−3^). *AP3D1* does not (*p* = 0.024). Both genes fall short of it at the broad endpoint (G6_EPLEPSY, 15,645 cases). Endpoint definitions follow FinnGen R12 registry codes, GE for generalised epilepsy and G6_EPLEPSY for all epilepsy. The direction of effect was concordant with ILAE3 in all four tests, and a formal test confirms that the effect sizes are compatible. Heterogeneity between the ILAE3 and FinnGen estimates is not significant at any comparison, with Cochran Q *p* between 0.39 and 0.57, *I* ^2^ = 0 (Supplementary Table S16). Colocalisation does not reproduce in FinnGen. The highest posterior obtained is 0.348 for *KCNIP2* at the generalised-epilepsy endpoint, well below the 0.8 threshold. FinnGen supports the direction and magnitude of the causal estimate but does not independently replicate the shared-variant inference. The difference reflects the smaller Finnish case count and different registry ascertainment, not a contradiction of the ILAE3 result.

### 3.4 Cell-type resolution can determine detectability

The *KCNIP2* colocalisation was confined to inhibitory neurons (Figure 5; Supplementary Figure S1). In bulk cortex, *KCNIP2* again carried a strong cis-eQTL, but the posterior for two distinct causal variants reached 1.00 over 2,656 shared variants, and the shared-variant posterior decreased to zero. The bulk-cortex eQTL lead (rs80080632) differed from the inhibitory-neuron lead (rs10883695) and was in negligible linkage disequilibrium with it, at *r* ^2^ = 0.016 in the European reference (Supplementary Table S17). The inhibitory-neuron lead carried no detectable effect on *KCNIP2* expression in bulk cortex (*β* = −0.018, *p* = 0.56). In excitatory neurons, *KCNIP2* carried a cis-eQTL that fell just short of genome-wide significance, on a different variant from the inhibitory lead, and did not colocalise (posterior 0.006). The full glial summary statistics show that *KCNIP2* is tested in all four glial panels. Three carry no cis-eQTL reaching the panel discovery threshold. Oligodendrocytes carry one (regional lead *F* = 21.6, *p* = 3.3 × 10^−6^), whose colocalisation posterior of 0.526 falls below the threshold. Per-panel effects and posteriors are given in Supplementary Table S18. Among single-nucleus panels, *KCNIP2* colocalised above the threshold only in inhibitory neurons (Supplementary Table S19). The colocalising signal at 10q24.32 is the main, sex-consistent generalised-epilepsy signal, with its epilepsy-association lead rs3740422 in near-complete linkage disequilibrium with the meta-analysis lead rs11191156 (*r* ^2^ = 0.975) and distinct from the separate female-specific signal at the locus (lead rs72845653, *r* ^2^ ≈ 0.03). *AP3D1*, in contrast, stayed above the colocalisation threshold in all three panels tested.

**FIGURE 5:**
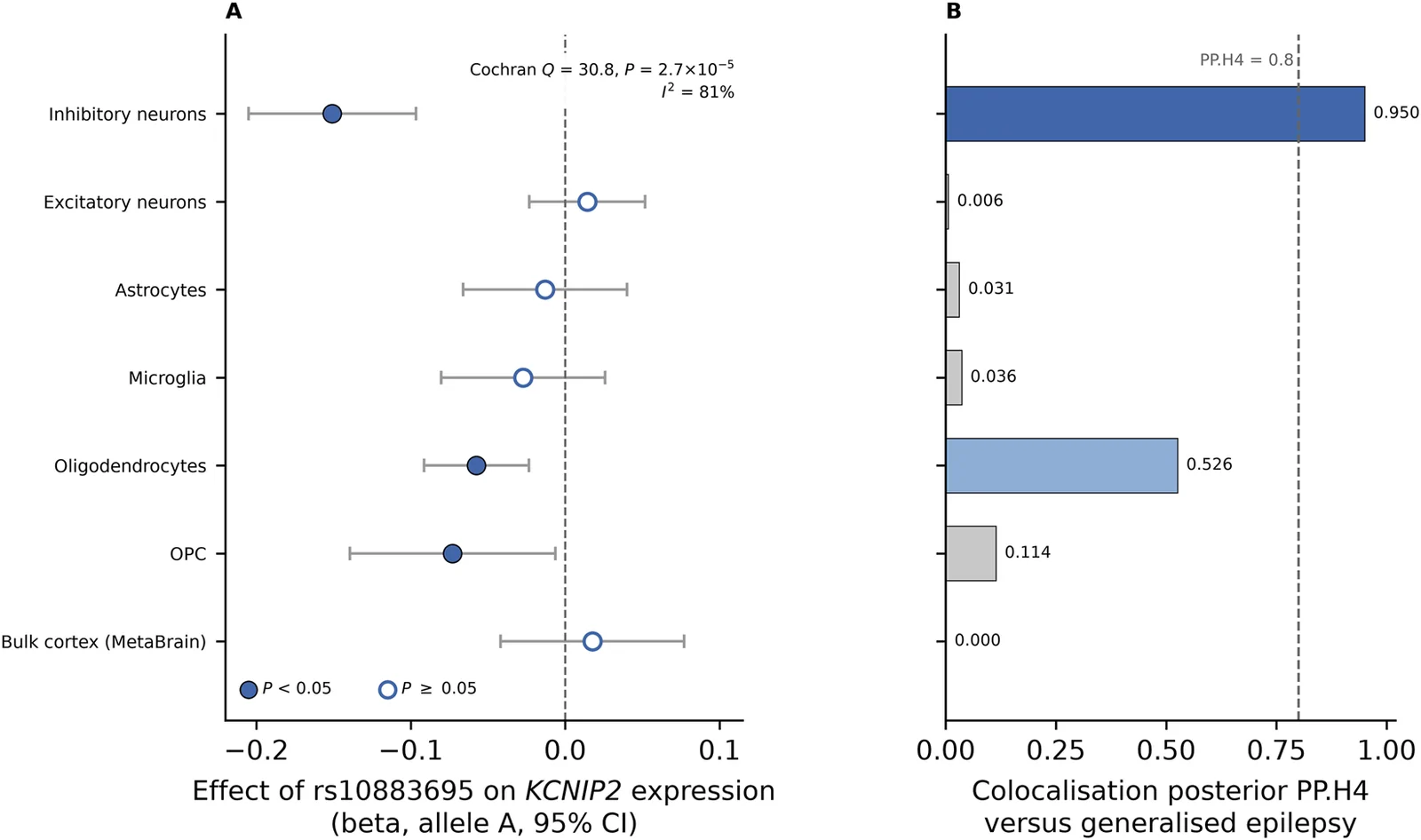
Cell-type-resolved effect and colocalisation at *KCNIP2*. **(A)** Effect of rs10883695, the inhibitory-neuron eQTL lead, on *KCNIP2* expression. This is shown in each of the seven panels with its 95% confidence interval. All values are aligned to allele. **(A)**. Filled markers denote *p* < 0.05. The cross-panel heterogeneity test is inset. **(B)** Colocalisation posterior against generalised epilepsy for the same panels. The 0.8 threshold is marked. Panels are distinct cell populations rather than ordered levels of one measurement. No connecting line is drawn. Astrocyte, microglial, oligodendrocyte, and progenitor values are computed from the full SingleBrain summary statistics.

### 3.5 Colocalisation rejects most MR associations

The colocalisation-first design separated the two retained genes from a larger set of MR associations (Figure 6). Eighteen of the 23 screened genes carried a testable cis instrument, one fewer than the 19 colocalisation-tested genes since *FAM19A5* had a cis-eQTL sufficient for colocalisation but no genome-wide instrument for MR. Across these, forward MR with multiple-testing correction returned 10 with a significant effect on epilepsy in at least one phenotype. Colocalisation retained only *KCNIP2* and *AP3D1* and rejected the other eight. Among these eight, three carried a regional disease signal resolving to a variant distinct from the expression lead (*CDK5RAP3*, *PCDH7*, and *RMI1*) and four had no regional disease signal able to support colocalisation (*CACNA2D2*, *KCNN2*, *POU3F3*, and *PTPRK*). *SCN8A*, the eighth, reached MR significance at all epilepsy, where its shared-variant posterior was 0.669, below the 0.8 threshold, and resolved to two distinct causal variants at generalised epilepsy. None of the eight reached the 0.8 shared-variant posterior in either phenotype. The 16 genes with a testable cis instrument that did not colocalise in inhibitory neurons also failed to colocalise in excitatory neurons, with two carrying a distinct excitatory neuron eQTL variant at a posterior above 0.98 for two causal variants rather than a shared one. The single-nucleus screen, therefore, returns concordant negatives across both neuronal panels rather than resolution-specific false positives. The strongest excitatory cis variant for *CHRM3* is rs184596375 (*β* = +0.134, SE = 0.023, *F* = 34.0, *p* = 5.5 × 10^−9^), against *F* = 11.6 for the best inhibitory-neuron variant (Supplementary Table S20). On the instrument used, rs12041407 (*F* = 20.2), *CHRM3* shows no causal effect. The OR is 0.77 (95% CI 0.37 to 1.59, *p* = 0.48) at generalised epilepsy and 0.91 (0.59–1.41, *p* = 0.69) at all epilepsy (Supplementary Table S21). Colocalisation is likewise negative in excitatory neurons, at posteriors of 0.007 for generalised epilepsy and 0.005 for all epilepsy across roughly 3,950 shared variants. The posterior mass sits instead on an expression-only signal (PP.H1 = 0.66 for generalised epilepsy and 0.65 for all epilepsy), with 0.26 on no signal in either trait. The region carries a clear cis-eQTL and no detectable epilepsy association rather than two distinct causal variants (PP.H3 = 0.055 and 0.064). *CHRM3* is, therefore, not supported as a causal gene in either neuronal class.

**FIGURE 6:**
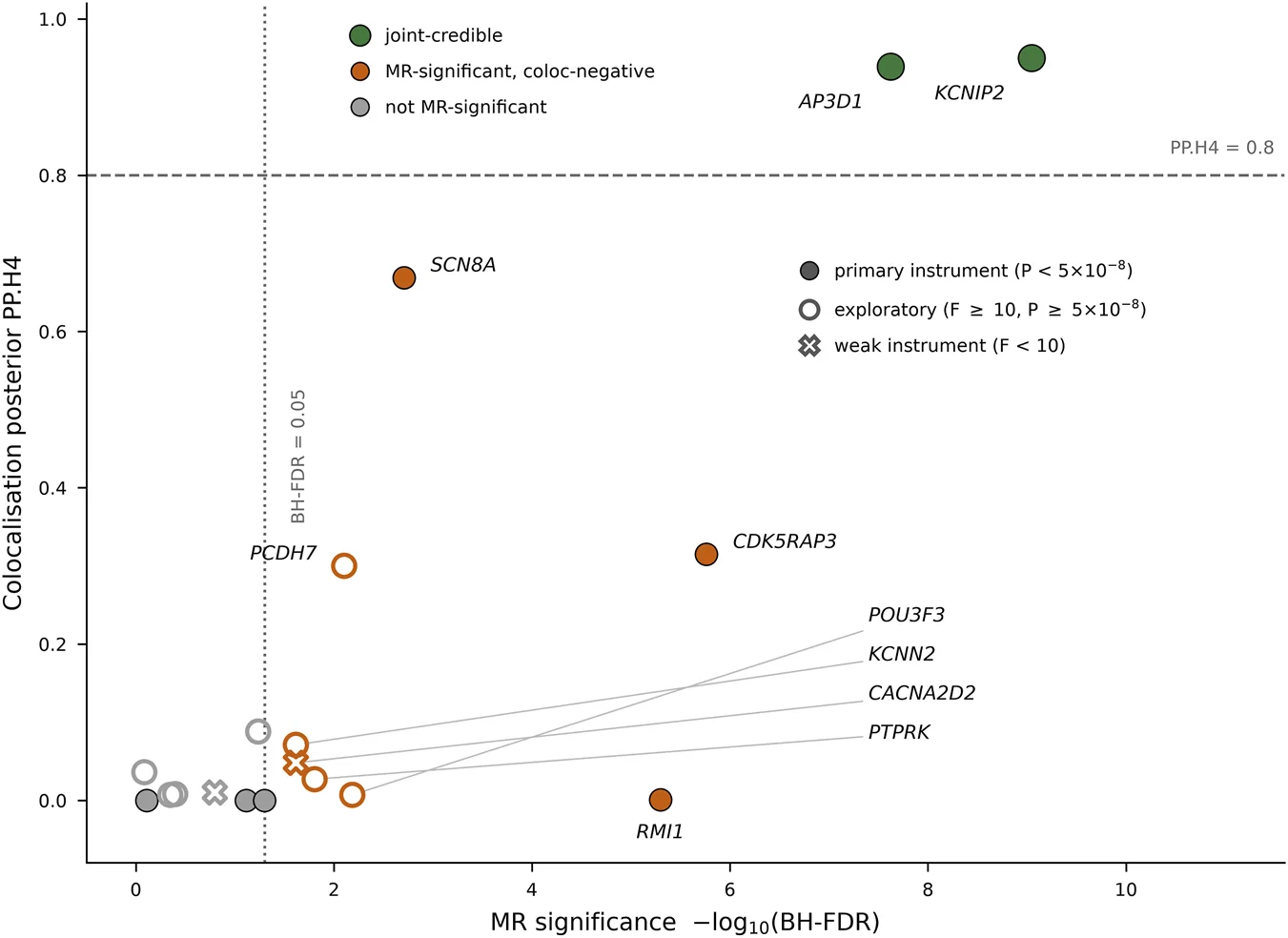
Colocalisation rejects most MR associations. Each tested gene is plotted by its inhibitory-neuron colocalisation posterior (PP.H4) on the y-axis against its MR significance (-log_10_ of the Benjamini–Hochberg FDR) on the x-axis. Both values are taken at the phenotype where the gene reaches MR significance, which is genetic generalised epilepsy except for *SCN8A* and *POU3F3*, shown at all epilepsy. Joint-credible genes pass both thresholds (PP.H4 > 0.8 and FDR <0.05) and comprise only *KCNIP2* and *AP3D1*. Genes that are MR-significant but colocalisation-negative pass the FDR threshold alone. Marker outline encodes instrument strength. Filled markers are primary instruments reaching *p* < 5 × 10^−8^, and open circles are exploratory instruments meeting *F* ≥ 10 only. Crosses are the two weak instruments with *F* < 10 (*CACNA2D2* and *BCL11A*), whose estimates are not interpretable. Eight genes carry a primary instrument, where eight are exploratory and two are weak.

Every *F-*value was re-derived from the raw association files. The *RBFOX1* inhibitory-neuron *F* is 26.7 for both the panel lead and the MR instrument. No gene reaches the 0.8 threshold in excitatory neurons, the highest being *CDK5RAP3* at 0.653 and every value reproduced in Supplementary Table S22. Five genes return a higher excitatory than the inhibitory posterior, *CDK5RAP3* 0.653 against 0.315, and *SCN8A*, *GLS*, *RYR2*, and *SCN1A* on much smaller values. Only *KCNIP2* crosses the threshold between resolutions, while several genes shift below it. The full comparison is shown in Supplementary Table S23.

Of the 10 MR-significant genes, five fail the primary instrument threshold. Four rest on exploratory instruments meeting *F* ≥ 10 only (*PCDH7*, *KCNN2*, *PTPRK*, and *POU3F3*), and *CACNA2D2* rests on a weak instrument below *F* = 10. The count is not stable under the stricter rule. Restricting the family to the eight genes with a primary instrument and recomputing the correction returns six MR-significant genes (*KCNIP2*, *AP3D1*, *CDK5RAP3*, *RMI1*, *RYR2*, and *SCN8A*) instead of 10. *RYR2*, not significant in the full family, becomes significant because the smaller family carries a lighter correction. *KCNIP2* and *AP3D1* hold their position under either rule, with instruments at *F* = 29.8 and *F* = 30.1 and MR *p-*values of 2.5 × 10^−11^ and 1.3 × 10^−9^ at generalised epilepsy. The colocalisation result is unchanged by the instrument rule (Supplementary Table S24).

### 3.6 Sensitivity analyses confirm the two retained colocalisations

The two retained colocalisations were stable. Both remained above 0.8 across the shared-variant prior from 5 × 10^−6^ to 1 × 10^−4^ and fell below the threshold only at the most extreme prior of 1 × 10^−6^, where the shared-variant hypothesis retained the highest posterior (Supplementary Figure S2). Steiger filtering supported the direction from expression to epilepsy for both genes. The SuSiE-based second method agreed with the approximate Bayes factor result, returning a single colocalising credible set per gene at posteriors of 0.89 for *KCNIP2* and 0.88 for *AP3D1*, with leads matching the discovery analysis (Supplementary Table S25). *GM2A* did not colocalise with the generalised or all-epilepsy signal at posteriors below 0.05 (Supplementary Table S26). The *ACE* positive control recovered its known strong plasma cis-pQTL and, mapping to no epilepsy locus, did not colocalise, so the *GM2A* null reflects the absence of a shared variant and not a failure of the plasma instrument. There is one expression signal and one disease signal at 10q24.32, one and one at 19p13.3, and two expression signals at *SCN8A*. *SCN8A* is the locus where the masked analysis matters most (Supplementary Table S27). *CUEDC2* returns no genome-wide significant expression signal of its own. Splitting the reference changes the *KCNIP2*–*CUEDC2* correlation from 0.975 to 0.982 and 0.967. It changes the correlation between the inhibitory-neuron and bulk leads from 0.016 to 0.035 and 0.004. The near-complete linkage between the two genes and the independence of the bulk signal both remained in the reference sample is used (Supplementary Table S28). Both loci returned a single credible set. The pipeline separates the extremes of its own range on the same code path, returning 0.939 for *AP3D1* against generalised epilepsy and 0.0001 for *RYR2*. *RYR2* is a negative control of a specific kind. It carries a strong inhibitory-neuron cis-eQTL (rs10925297, *F* = 47.2, *p* = 6.3 × 10^−12^), and the region also carries an epilepsy association. The two map to different variants, so the posterior concentrates on distinct causal variants (PP.H3 = 0.998).

The single-causal-variant assumption was tested by masking the primary shared peak (Supplementary Table S29). No secondary colocalisation appears. For the four genes with regional data, *SCN8A*, *KCNN2*, *CACNA2D2*, and *POU3F3*, the masked posterior for a shared variant decreases in every case. It falls from 0.669 to 0.002 for *SCN8A* at all epilepsy and from 0.104 to 0.010 for *KCNN2*. *CACNA2D2* falls from 0.048 to 0.016 and *POU3F3* from 0.021 to 0.005. In each case, the residual mass moves onto the no-signal hypothesis. *SCN8A* at generalised epilepsy gives a decisive posterior for distinct causal variants (0.994). *KCNN2* at generalised epilepsy is weaker at 0.426 and does not reach a threshold supporting that reading alone. *CACNA2D2*, *POU3F3*, and *PTPRK* return low posteriors for every hypothesis, including the null. For *SCN8A* at all epilepsy, the unmasked posterior of 0.669 approaches the threshold without reaching it.

## 4 Discussion

Whether a druggable epilepsy-locus gene is detectable can depend on the cell type in which its expression is measured. *KCNIP2* shows this in the present study. At the 10q24.32 locus, bulk cortex points to distinct causal variants, with the shared-variant posterior at zero and the bulk and neuronal leads in negligible linkage disequilibrium, so a bulk or plasma analysis discards the gene. The shared signal appears only at inhibitory-neuron resolution. The neuronal signal is, therefore, not so much diluted in bulk as replaced at the lead by a separate variant, the pattern tissue averaging predicts, and this offers a mechanism for the weak and inconsistent results of earlier analyses. The opposite reading, that the bulk result is correct and the neuronal colocalisation is the artefact, is countered by the directional replication in an independent biobank, the stability across colocalisation priors, and the agreement of a second method, though single-nucleus data are noisier than bulk and the demonstration rests on one gene.

Only one gene in the screen behaves this way. Of the 18 genes with a testable cis instrument, *KCNIP2* was the only one whose colocalisation changed between resolutions. The 16 genes that did not colocalise in inhibitory neurons also did not colocalise in excitatory neurons. *AP3D1* colocalised in every panel tested at generalised epilepsy (0.94 in inhibitory neurons, 0.94 in excitatory neurons, and 0.92 in bulk cortex). It colocalised in none of them at all epilepsy (0.29, 0.25, and 0.08). Its pattern is, therefore, set by the phenotype rather than by resolution. Single-nucleus resolution, therefore, did not manufacture colocalisations, and it did not lose the one that bulk cortex also detected. The screen cannot show how common this pattern is. Colocalisation has power only at genome-wide significant loci, and only 23 genes were available. Cell-type-restricted candidates have been reported in ischaemic stroke and in migraine (Seo et al., 2024; Ye et al., 2025). *KCNIP2* is a proof of principle, not a general property of epilepsy loci.

Cell-type restriction was tested formally, not inferred from which panels reached significance. Detection in one panel and absence in another can reflect panel power (Yu et al., 2025). The test used the full glial summary statistics of the SingleBrain resource (Jang et al., 2026). It did not use the per-gene top-association files. *KCNIP2* is tested in all four glial panels. In the per-gene top-association files, it falls below each panel’s own discovery threshold. This reflects the threshold and not an absence of data. At rs10883695, the inhibitory-neuron lead, the effect on *KCNIP2* expression is −0.151 (SE 0.028, *p* = 4.9 × 10^−8^) in inhibitory neurons. Aligned to the same allele, it is +0.014 (SE 0.019, *p* = 0.45) in excitatory neurons. It is −0.013 (SE 0.027, *p* = 0.63) in astrocytes, −0.027 (SE 0.027, *p* = 0.32) in microglia, −0.057 (SE 0.017, *p* = 4.3 × 10^−5^) in oligodendrocytes, and −0.073 (SE 0.034, *p* = 0.032) in oligodendrocyte progenitors. In bulk cortex, it is +0.018 (SE 0.030, *p* = 0.56). The variant has a minor allele frequency of 0.278 in the 1000 Genomes European reference. Cochran’s Q across the seven panels is 30.8 on six degrees of freedom (*p* = 2.7 × 10^−5^, *I* ^2^ = 80.5%). Pairwise contrasts of every panel against inhibitory neurons are given in Supplementary Table S30.

A reciprocal test repeats the comparison from each panel’s own lead variant. This confirms the heterogeneity is not an artefact of testing the inhibitory-neuron lead, as shown in Supplementary Table S31. Against inhibitory neurons, the difference is significant for excitatory neurons (−0.165, 95%t CI -0.231 to −0.099, *p* = 9.0 × 10^−7^). It is significant for astrocytes (−0.138, *p* = 3.7 × 10^−4^) and microglia (−0.124, *p* = 1.4 × 10^−3^), oligodendrocytes (−0.094, *p* = 4.2 × 10^−3^), and bulk cortex (−0.169, *p* = 4.0 × 10^−5^), but it is not significant for progenitors, the smallest panel (−0.078, *p* = 0.075). The four glial panels are mutually consistent (Q = 3.0, *p* = 0.39, *I* ^2^ = 0.6%). The heterogeneity is carried by inhibitory neurons against the rest, not distributed across cell types. The claim of exclusive inhibitory neuron regulation is narrowed accordingly. Oligodendrocytes carry a genuine cis-eQTL for *KCNIP2*, with a regional lead at *F* = 21.6 (*p* = 3.3 × 10^−6^). Its colocalisation posterior against generalised epilepsy is 0.526, below the 0.8 threshold. This is well above the values in astrocytes (0.031), microglia (0.036), progenitors (0.114), excitatory neurons (0.006), and bulk cortex (0.000). The effect in oligodendrocytes runs in the same direction as in inhibitory neurons, at roughly a third of its size.

The disease-colocalising signal is the strongest in inhibitory neurons. A weaker, directionally concordant and sub-threshold signal appears in oligodendrocytes. Regulation is not unique to one cell type. The instrument rule also bounds the MR layer. A drug-target design draws its strength from the cis window of a single gene (Schmidt et al., 2020). Ten of the 18 instruments fail to reach genome-wide significance in the inhibitory-neuron panel. In each case, the gene has no genome-wide significant cis-eQTL there. This shortfall reflects the power of the eQTL panel, not the choice of variant within it. Estimates from these genes are exploratory. The count of MR nominations should be read as a property of the admission rule, not as a fixed number (Burgess et al., 2016).

The inhibitory-neuron signal has other possible explanations. Statistical power is one of them. Single-nucleus panels are smaller and less well powered than bulk studies (Yu et al., 2025), and a colocalisation posterior also depends on the assumption of a single causal variant (Wallace, 2021). A low posterior in one panel, therefore, does not mean the regulatory effect is absent there. Three of the four glial panels carried no *KCNIP2* cis-eQTL reaching the panel discovery threshold, which is an absence of evidence. Oligodendrocytes carried a genuine cis-eQTL, whose colocalisation posterior of 0.526 falls below the 0.8 threshold. Three of the four glial panels carried no KCNIP2 ciseQTL. Glial mechanisms contribute to seizures through other routes (Patel et al., 2019). Different tissues may also capture different regulatory biology instead of a diluted version of the same signal. Bulk tissue loses cell-type signal through cellular heterogeneity (Yu et al., 2025), but it also recovers genes that cell-type analyses miss (Liu et al., 2025a). In bulk cortex, *KCNIP2* carries a strong cis-eQTL at a different lead variant (rs80080632, *β* = −0.381, *p* = 3.3 × 10^−10^). That variant has no effect in inhibitory neurons (*β* = −0.009, *p* = 0.88). It is associated with expression in excitatory neurons (*β* = −0.183, SE = 0.040, *p* = 5.7 × 10^−6^). The two resolutions are, therefore, measuring different regulatory events, not one diluting the other (Supplementary Table S32). Bulk is not missing the neuronal signal. It is dominated by a separate one that does not colocalise with epilepsy.

A variant can also be detected in one cell type while acting on excitability in another. The colocalising eQTL is measured in inhibitory neurons, whereas KChIP2 regulation of the A-type current has been characterised mainly in excitatory neurons, particularly the dendrites of hippocampal pyramidal cells (Murphy and Hoffman, 2019). The phenotype matters as well. *AP3D1* colocalised for generalised epilepsy but not for all epilepsy, so the trait analysed also changes what is detected.

This dependence on cell type helps explain why plasma proteome MR (Fu et al., 2025; Gui et al., 2025) and multi-scale eQTL integration (Zhong et al., 2026) have prioritised largely different genes. The multi-scale analysis is the closest in method to the present work, since it too draws on single-cell and bulk eQTL. It combines summary-data-based MR with colocalisation, whereas this study retains a gene only when a shared causal variant is supported. Its leading candidate FGFR3 is expressed mainly in astrocytes, and its second candidate HAGH is expressed in neurons, and neither gene appears among the candidates from the plasma studies or from this study. A screen at a single resolution, often a non-neuronal one, can recover signals that are not the neuronal ones driving disease. Matching the cell type can, therefore, change which gene is selected, at least at loci of this kind. *AP3D1* sets a limit on this argument, since its expression colocalises in all three panels tested for genetic generalised epilepsy, though not for all epilepsy, and reproduces in an independent dataset. For this gene, the choice of cell type does not change whether the signal is detected. A colocalisation of that strength is still not a therapeutic hypothesis. Bi-allelic loss of *AP3D1* function causes a severe neurodevelopmental disorder with seizures and defines Hermansky–Pudlak syndrome type 10 (Ammann et al., 2016), whereas the common-variant effect estimated in this study links higher expression to higher risk and reproduces.

These opposite directions have two possible explanations. One is a non-monotonic relationship between *AP3D1* dosage and seizure liability across the range from bi-allelic loss to common variation. The other is that the rare loss-of-function and the common regulatory effects act through separate mechanisms. *AP3D1* is, therefore, a supported epilepsy association, but its direction of effect does not translate into a therapeutic hypothesis. The locus at 10q24.32 does not resolve to a single gene. The colocalisation supports one shared regulatory variant in inhibitory neurons. That variant is also the strongest cis-eQTL variant for the neighbouring *CUEDC2*, and the two are in near-complete linkage disequilibrium (*r* ^2^ = 0.975).

Conditioning on this variant removed the epilepsy association for both genes. The genetic evidence, therefore, identifies a shared *KCNIP2* and *CUEDC2* regulatory locus, not a single implicated gene. Three points favour *KCNIP2*. Only *KCNIP2* has a cis-eQTL that reaches genome-wide significance. Its expression effect is the larger of the two. The Kv4-KChIP2 channel links reduced *KCNIP2* expression to higher excitability (An et al., 2000; Wang et al., 2013). *CUEDC2* cannot be excluded.

The two genes also differ in pharmacological tractability (Supplementary Table S33). *KCNIP2* encodes the EF-hand accessory subunit KChIP2 of the Kv4 A-type potassium channel, and the Kv4-KChIP2 complex is a structurally resolved target (Protein Data Bank 7UKH and 7W6S, ChEMBL CHEMBL3885598). KChIP2 raises the Kv4 A-type potassium current (An et al., 2000), and its deletion reduces that current, increases neuronal excitability, and raises kindling-induced seizure susceptibility in mice (Wang et al., 2013). The protective direction estimated in this study is the one this biology predicts, and human genetics implicates the same channel from the pore-forming side, since a *de novo* variant in *KCND2*, which encodes the Kv4.2 subunit that KChIP2 modulates, alters A-type-current inactivation and causes intractable seizures (Lee et al., 2014). A knock-in mouse carrying that variant develops spike-wave epileptiform discharges (Jerng et al., 2026).

Positive modulation of the A-type current is, therefore, the candidate on-target therapeutic direction, which makes *KCNIP2* a tractable target class but not a validated target. KChIP2 also regulates the equivalent potassium current in cardiac muscle, so a strategy that potentiates it carries an on-target cardiac liability (Liu et al., 2025b). KChIP2 is one of several closely related subunits (KChIP1, KChIP3, KChIP4, DPP6, and DPP10), so selective targeting is a further difficulty. *AP3D1* encodes a subunit of the intracellular AP-3 vesicle-trafficking adaptor, a protein class with no catalytic ligand pocket and, therefore, low small-molecule tractability. This low tractability and the uninterpretable common-variant direction leave *AP3D1* as a supported epilepsy gene but a weak drug-target candidate. The tractability comparison sets the scope of what this study claims, which is to prioritise loci and not to deliver validated drug targets. That genetic effects on expression are tissue- and cell-type-dependent is established (Consortium et al., 2020), and genes at epilepsy loci have already been prioritised (ILAE Consortium on Complex Epilepsies) (Stevelink et al., 2023). The contribution of this study is to apply that principle to epilepsy at cell-type resolution. It measures directly what bulk and plasma analyses miss and uses a colocalisation filter that separates two supported candidates from a larger set of MR associations.

This study has several limitations. The demonstration that the resolution determines detectability rests mainly on the single gene *KCNIP2*. The discovery space is also small, since colocalisation has power only at genome-wide significant loci and the generalised-epilepsy case count is modest, so widening it requires a larger epilepsy GWAS. The two colocalisations were not replicated independently because the largest generalised-epilepsy resource was used in discovery and the available biobank is underpowered at these loci, so replication is directional and not a second colocalisation. No functional work was performed, so the causal role of the two genes in seizure liability remains to be established. Three narrower points qualify the interpretation. *KCNIP2* cannot be separated from its neighbour *CUEDC2* on genetic evidence alone. The nomination of *KCNIP2* rests on its cis-eQTL reaching genome-wide significance, where that of *CUEDC2* does not (Supplementary Table S34), and on the established Kv4-KChIP2 channel biology, not on the colocalisation. The colocalising *KCNIP2* eQTL is measured in inhibitory neurons, whereas the established A-type-current action of KChIP2 is measured in excitatory neurons, so the cell type in which the variant is detectable differs from the one through which it acts. The bulk and single nucleus eQTL also differ in cohort and platform, although a distinct bulk lead variant that carries no neuronal signal places this difference at the causal variant and not in dataset noise. Finally, 10q24.32 carries the discovery study’s one female-specific signal, with opposite direction in females and males; the signal analysed here is the main, sex-consistent association, but the protective estimate is from sex-combined data, and a sex-stratified analysis would be needed before generalising it across sexes.

## 5 Conclusion

In this study, a cell-type-resolved colocalisation screen shows that the cell type can determine whether an epilepsy drug-target signal is detectable. *KCNIP2* shows this most clearly, since its inhibitory-neuron expression colocalised with generalised epilepsy in a protective direction; yet, in bulk cortex, the same signal is lost because the disease and the expression come from different variants. A bulk or plasma screen misses it. Colocalisation also rejected most MR-significant genes, and an earlier plasma-proteome candidate again did not colocalise. At this locus, single-cell resolution is what made the signal detectable. That is a proof of principle for cell-type-resolved screening, not a general rule for epilepsy loci. *KCNIP2* is a candidate target found at inhibitory-neuron resolution**,** although the shared signal does not separate it from *CUEDC2*. It needs fine-mapping and functional study. *AP3D1* is a genetically supported epilepsy gene but a pharmacologically intractable target.

## Data Availability

The original contributions presented in the study are included in the article/Supplementary Material; further enquiries can be directed to the corresponding author.

## References

[B1] Ammann S. Schulz A. Krägeloh-Mann I. Dieckmann N. M. Niethammer K. Fuchs S. (2016). Mutations in AP3D1 associated with immunodeficiency and seizures define a new type of hermansky-pudlak syndrome. Blood 127, 997–1006. 10.1182/blood-2015-09-671636 26744459 PMC7611501

[B2] An W. F. Bowlby M. R. Betty M. Cao J. Ling H. P. Mendoza G. (2000). Modulation of A-type potassium channels by a family of calcium sensors. Nature 403, 553–556. 10.1038/35000592 10676964

[B3] Auton A. Brooks L. D. Durbin R. M. Garrison E. P. Kang H. M. Korbel J. O. (2015). A global reference for human genetic variation. Nature 526, 68–74. 10.1038/nature15393 26432245 PMC4750478

[B4] Benjamini Y. Hochberg Y. (1995). Controlling the false discovery rate: a practical and powerful approach to multiple testing. J. R. Stat. Soc. Ser. B Methodol. 57, 289–300. 10.1111/j.2517-6161.1995.tb02031.x

[B5] Bryois J. Calini D. Macnair W. Foo L. Urich E. Ortmann W. (2022). Cell-type-specific cis-eQTLs in eight human brain cell types identify novel risk genes for psychiatric and neurological disorders. Nat. Neurosci. 25, 1104–1112. 10.1038/s41593-022-01128-z 35915177

[B6] Burgess S. Dudbridge F. Thompson S. G. (2016). Combining information on multiple instrumental variables in Mendelian randomization: comparison of allele score and summarized data methods. Stat. Med. 35, 1880–1906. 10.1002/sim.6835 26661904 PMC4832315

[B7] Consortium T. G. Aguet F. Anand S. Ardlie K. G. Gabriel S. Getz G. A. (2020). The GTEx consortium atlas of genetic regulatory effects across human tissues. Science 369, 1318–1330. 10.1126/science.aaz1776 PMC7737656 32913098

[B8] De Klein N. Tsai E. A. Vochteloo M. Baird D. Huang Y. Chen C. Y. (2023). Brain expression quantitative trait locus and network analyses reveal downstream effects and putative drivers for brain-related diseases. Nat. Genet. 55, 377–388. 10.1038/s41588-023-01300-6 36823318 PMC10011140

[B9] Ferkingstad E. Sulem P. Atlason B. A. Sveinbjornsson G. Magnusson M. I. Styrmisdottir E. L. (2021). Large-scale integration of the plasma proteome with genetics and disease. Nat. Genet. 53, 1712–1721. 10.1038/s41588-021-00978-w 34857953

[B10] Finan C. Gaulton A. Kruger F. A. Lumbers R. T. Shah T. Engmann J. (2017). The druggable genome and support for target identification and validation in drug development. Sci. Transl. Med. 9, eaag1166. 10.1126/scitranslmed.aag1166 28356508 PMC6321762

[B11] Fu J. Wu W. Shen S. (2025). Assessing the causal relationship between the plasma proteome and epilepsy: a Mendelian randomization study. Epilepsia Open 10, 1966–1977. 10.1002/epi4.70175 41165052 PMC12716302

[B12] Fujita M. Gao Z. Zeng L. Mccabe C. White C. C. Ng B. (2024). Cell subtype-specific effects of genetic variation in the alzheimer’s disease brain. Nat. Genet. 56, 605–614. 10.1038/s41588-024-01685-y 38514782 PMC12288883

[B13] Gabitto M. I. Travaglini K. J. Rachleff V. M. Kaplan E. S. Long B. Ariza J. (2024). Integrated multimodal cell atlas of Alzheimer’s disease. Nat. Neurosci. 27, 2366–2383. 10.1038/s41593-024-01774-5 39402379 PMC11614693

[B14] Giambartolomei C. Vukcevic D. Schadt E. E. Franke L. Hingorani A. D. Wallace C. (2014). Bayesian test for colocalisation between pairs of genetic association studies using summary statistics. PLoS Genet. 10, e1004383. 10.1371/journal.pgen.1004383 24830394 PMC4022491

[B15] Gui J. Chu H. Zhang J. Li X. Liu W. Li R. (2025). Proteome-wide and immune cell phenotype mendelian randomization highlights immune involvement in genetic generalized epilepsy. Brain Behav. 15, e70625. 10.1002/brb3.70625 40495522 PMC12152256

[B16] Hemani G. Tilling K. Davey Smith G. (2017). Orienting the causal relationship between imprecisely measured traits using GWAS summary data. PLoS Genet. 13, e1007081. 10.1371/journal.pgen.1007081 29149188 PMC5711033

[B17] Jang B. Bp K. Tokolyi A. Dredge W. H. Ravi A. Jung S. H. (2026). A meta-analysis of single-nucleus expression quantitative trait loci linking genetic risk to brain disorders. Nat. Genet. 58, 737–747. 10.1038/s41588-026-02541-x 41857317

[B18] Jerng H. H. Silva-Pérez M. David L. S. Chin J. Krishnan V. Pfaffinger P. J. (2026). Kv4.2(V404M) mutation induces epileptiform activity and multiple behavioral abnormalities in heterozygous Knock-in mice. J. Neurosci. 46, e1256252026. 10.1523/jneurosci.1256-25.2026 41554648 PMC12896680

[B19] King E. A. Davis J. W. Degner J. F. (2019). Are drug targets with genetic support twice as likely to be approved? Revised estimates of the impact of genetic support for drug mechanisms on the probability of drug approval. PLoS Genet. 15, e1008489. 10.1371/journal.pgen.1008489 31830040 PMC6907751

[B20] Kurki M. I. Karjalainen J. Palta P. Sipilä T. P. Kristiansson K. Donner K. M. (2023). FinnGen provides genetic insights from a well-phenotyped isolated population. Nature 613, 508–518. 10.1038/s41586-022-05473-8 36653562 PMC9849126

[B21] Lee H. Lin M. C. Kornblum H. I. Papazian D. M. Nelson S. F. (2014). Exome sequencing identifies *de novo* gain of function missense mutation in KCND2 in identical twins with autism and seizures that slows potassium channel inactivation. Hum. Mol. Genet. 23, 3481–3489. 10.1093/hmg/ddu056 24501278 PMC4049306

[B22] Li X. Shen A. Zhao Y. Xia J. (2023). Mendelian randomization using the druggable genome reveals genetically supported drug targets for psychiatric disorders. Schizophr. Bull. 49, 1305–1315. 10.1093/schbul/sbad100 37418754 PMC10483453

[B23] Liao C. C. Wu S. A. Lee C. I. Liao K. R. Li J. M. (2025). Investigating causal relationships between gene expression and major depressive disorder *via* brain bulk-tissue and cell type-specific eQTL: a Mendelian randomization and Bayesian colocalization study. J. Affect Disord. 383, 167–178. 10.1016/j.jad.2025.04.161 40311809

[B24] Liu S. Cho M. Huang Y. N. Park T. Chaudhuri S. Rosewood T. J. (2025a). Multi-omics analysis for identifying cell-type-specific and bulk-level druggable targets in alzheimer's disease. J. Transl. Med. 23, 788. 10.1186/s12967-025-06739-1 40653482 PMC12257706

[B25] Liu W. Liu J. Wang G. Cheng W. Gong H. Song Y. (2025b). Down-regulation of histone deacetylase 2 attenuates ventricular arrhythmias in a mouse model of cardiac hypertrophy through up-regulation of Kv channel-interacting protein 2 expression. Cardiovasc Res. 121, 424–439. 10.1093/cvr/cvaf008 39870585

[B26] Löscher W. Potschka H. Sisodiya S. M. Vezzani A. (2020). Drug resistance in epilepsy: clinical impact, potential mechanisms, and new innovative treatment options. Pharmacol. Rev. 72, 606–638. 10.1124/pr.120.019539 32540959 PMC7300324

[B27] Mathys H. Peng Z. Boix C. A. Victor M. B. Leary N. Babu S. (2023). Single-cell atlas reveals correlates of high cognitive function, dementia, and resilience to alzheimer's disease pathology. Cell 186, 4365–4385.e27. 10.1016/j.cell.2023.08.039 37774677 PMC10601493

[B28] Murphy J. G. Hoffman D. A. (2019). A polybasic motif in alternatively spliced KChIP2 isoforms prevents Ca(2+) regulation of Kv4 channels. J. Biol. Chem. 294, 3683–3695. 10.1074/jbc.RA118.006549 30622142 PMC6416424

[B29] Nelson M. R. Tipney H. Painter J. L. Shen J. Nicoletti P. Shen Y. (2015). The support of human genetic evidence for approved drug indications. Nat. Genet. 47, 856–860. 10.1038/ng.3314 26121088

[B30] Patel D. C. Tewari B. P. Chaunsali L. Sontheimer H. (2019). Neuron-glia interactions in the pathophysiology of epilepsy. Nat. Rev. Neurosci. 20, 282–297. 10.1038/s41583-019-0126-4 30792501 PMC8558781

[B31] Schmidt A. F. Finan C. Gordillo-Marañón M. Asselbergs F. W. Freitag D. F. Patel R. S. (2020). Genetic drug target validation using Mendelian randomisation. Nat. Commun. 11, 3255. 10.1038/s41467-020-16969-0 32591531 PMC7320010

[B32] Seo Y. Bae H. Lee C. (2024). Bayesian colocalization of GWAS and eQTL signals reveals cell type-specific genes and regulatory variants for susceptibility to subtypes of ischemic stroke. Comput. Biol. Chem. 110, 108086. 10.1016/j.compbiolchem.2024.108086 38744227

[B33] Stevelink R. Campbell C. Chen S. Abou-Khalil B. Adesoji O. M. Afawi Z. (2023). GWAS meta-analysis of over 29,000 people with epilepsy identifies 26 risk loci and subtype-specific genetic architecture. Nat. Genet. 55, 1471–1482. 10.1038/s41588-023-01485-w PMC10484785 37653029

[B34] Wallace C. (2021). A more accurate method for colocalisation analysis allowing for multiple causal variants. PLoS Genet. 17, e1009440. 10.1371/journal.pgen.1009440 34587156 PMC8504726

[B35] Wang H. G. He X. P. Li Q. Madison R. D. Moore S. D. Mcnamara J. O. (2013). The auxiliary subunit KChIP2 is an essential regulator of homeostatic excitability. J. Biol. Chem. 288, 13258–13268. 10.1074/jbc.M112.434548 23536187 PMC3650365

[B36] Wang G. Sarkar A. Carbonetto P. Stephens M. (2020). A simple new approach to variable selection in regression, with application to genetic fine mapping. J. R. Stat. Soc. Ser. B Stat. Methodol. 82, 1273–1300. 10.1111/rssb.12388 37220626 PMC10201948

[B37] Ye H. Huang Y. Wang C. Jin J. Jiang C. Fang J. (2025). Mapping the brain cell-specific regulatory architecture of migraine: a single-cell causal framework nominating inhibitory-neuronal BTBD16 and astrocytic RIMS1 as therapeutic targets. J. Headache Pain 27, 14. 10.1186/s10194-025-02241-7 41345547 PMC12797516

[B38] Yu X. Hu X. Wan X. Zhang Z. Wan X. Cai M. (2025). A unified framework for cell-type-specific eQTL prioritization by integrating bulk and scRNA-seq data. Am. J. Hum. Genet. 112, 332–352. 10.1016/j.ajhg.2024.12.018 39824189 PMC11866979

[B39] Zhong G. Y. Liu C. Wang H. L. Liang M. Liu Z. L. (2026). Multi-scale genetic and transcriptomic analyses identify druggable targets for epilepsy. Curr. Med. Sci. 46, 713–724. 10.1007/s11596-026-00194-9 42012806

[B40] Zuber V. Grinberg N. F. Gill D. Manipur I. Slob E. A. W. Patel A. (2022). Combining evidence from Mendelian randomization and colocalization: review and comparison of approaches. Am. J. Hum. Genet. 109, 767–782. 10.1016/j.ajhg.2022.04.001 35452592 PMC7612737

